# A case of hypokalemia and proteinuria with a new mutation in the SLC12A3 Gene

**DOI:** 10.1186/s12882-018-1083-2

**Published:** 2018-10-19

**Authors:** Qin Chen, Yaqin Wu, Jingya Zhao, Ying Jia, Wei Wang

**Affiliations:** 1 Department of Nephrology, Tongde Hospital of Zhejiang Province, Gucui Road, No.234, Hangzhou, 310012 Zhejiang People’s Republic of China; 2 Department of Internal Medicine, Cixi Chinese Medical Hospital, Cixi, 315300 Zhejiang People’s Republic of China; 3 Zhejiang Chinese Medical University, Hangzhou, 310053 Zhejiang People’s Republic of China

**Keywords:** Hypokalemia, Proteinuria, Mutation, SLC12A3 gene

## Abstract

**Background:**

Gitelman syndrome is an autosomal recessive inherited renal disorder characterized by hypokalemia, hypomagnesemia, and hypocalciuria. Since the symptoms are not severe and laboratory results are not always clear, Gitelman syndrome can go unnoticed by physicians. Here, we report our experiences with a patient that presented with hypokalemia and proteinuria; genetic analysis revealed a new homozygous mutation in the SLC12A3 gene.

**Case presentation:**

A 47-year-old man presented with hypokalemia and proteinuria. He had come to the hospital with the same symptoms 11 months and 3 years prior. His laboratory tests showed hypokalemia, hypocalciuria, and increased plasma angiotensin-2 activity. His renal pathology was consistent with the development of minimal lesions. Genetic analysis found a new homozygous mutation in exon 6 on the SLC12A3 gene (p.Trp281Arg) in the patient and in his brother; his mother and sister were diagnosed as heterozygous carriers of the same gene mutation. Finally, the patient was diagnosed with Gitelman syndrome.

**Conclusions:**

This case is the first to report a homozygous mutation in the 841th nucleotide of exon 6 on the SLC12A3 gene (p.Trp281Arg), which may cause Gitelman syndrome. At the same time, this report might stimulate interest in discussing the relationship between different mutations in the SLC12A3 gene and renal pathology.

## Background

Gitelman syndrome (GS) is a recessively inherited salt-losing tubulopathy characterized by hypomagnesemia, hypocalciuria, and secondary aldosteronism; this condition is responsible for hypokalemia and metabolic alkalosis [1]. With a prevalence of ∼1–10 per 40,000 persons and a potentially higher incidence in Asia, GS is one of the most frequently inherited tubular disorders [2]. GS is caused by mutations in the SLC12A3 gene coding for the thiazide-sensitive sodium-chloride co-transporter (NCCT), which is normally expressed in the first part of the distal convoluted tubule (DCT). At present, more than 350 mutations scattered throughout the SLC12A3 gene have been identified in GS patients [3]. These mutations include missense, nonsense, frame-shift, and splice-site mutations, which are distributed throughout the whole protein [1]. Here, we report our experiences with a patient that presented with hypokalemia and proteinuria; genetic analysis revealed a new homozygous mutation in the SLC12A3 gene.

## Case presentation

A 47-year-old man presented with hypokalemia and mild proteinuria owing to an unexplained syncope that occurred 8 years prior. He neglected his condition after taking oral medication to correct the hypokalemia. Three years ago, he manifested with severe proteinuria and was hospitalized in Shanghai Zhongshan Hospital. Serum creatinine (Cr) and albumin (alb) were 58 μmol/L and 22 g/L, respectively. Results from a 24 h urine protein excretion test detected 10.2 g of protein. Initial blood electrolytes, 24 h urine electrolytes, and the random UK/UCr ratio are shown in Table 1. Serological tests for infections and autoantibodies were negative. Physical examination, renal sonogram, and urogenital studies were normal. Neither hearing loss nor any ophthalmologic abnormalities were noted. He was diagnosed with nephrotic syndrome and a renal biopsy was performed.

**Table 1:** Biochemical index and patient medication

Date	Serum (mmol/L)						AII (pg/ml)	Urinary (mmol/24 h)					UK/UCr (mmol/g)	Medications		
	K (3.5–5.3)	Na (137–147)	Ca (2.15–2.55)	Mg (0.67–1.04)	Cl (99–110)	HCO3 (23–29)		K (25–100)	Na (130–260)	Ca (2.5–8)	Mg (2.5–8.5)	Cl (170–250)		KCl	Spironolactone	Magnesium Aspartate
13/06/04	3.2↓	145	1.99↓	0.92	108	26	ND	46.2	279↑	1.26↓	2.1↓	225.9	28.6↑	1.5 g/d	/	/
13/06/13	3.2↓	139	ND	ND	98↓	30↑	ND	ND	ND	ND	ND	ND	ND	1.5 g/d	/	/
16/04/10	3.29↓	140	2.47	0.92	100.7	25	High	93.61	144	0.8↓	1.88↓	161	57.6↑	2.5 g/d	/	/
16/05/05	3.37↓	138.9	2.09↓	1.08↑	99.2	28	ND	ND	ND	ND	ND	ND	ND	4 g/d	/	/
16/08/18	3.18↓	139.1	2.2	0.9	98.7↓	28	ND	ND	ND	ND	ND	ND	ND	4 g/d	/	/
16/10/27	3.46↓	137.2	2.28	0.86	98.7↓	27	ND	ND	ND	ND	ND	ND	ND	4 g/d	80 mg/d	/
16/11/04	3.46↓	139.8	2.25	0.92	102	26	ND	ND	ND	ND	ND	ND	ND	3.5 g/d	80 mg/d	140 mg/d
16/12/01	4.25	98.6↓	2.44	1.3↑	95↓	32↑	ND	ND	ND	ND	ND	ND	ND	3 g/d	80 mg/d	140 mg/d
17/01/08	3.86	100.4↓	2.26	0.95	100.4	30↑	ND	ND	ND	ND	ND	ND	ND	3 g/d	80 mg/d	140 mg/d

*AII* angiotensin-2, *ND* not determined, */* not taking

**Fig. 1:**
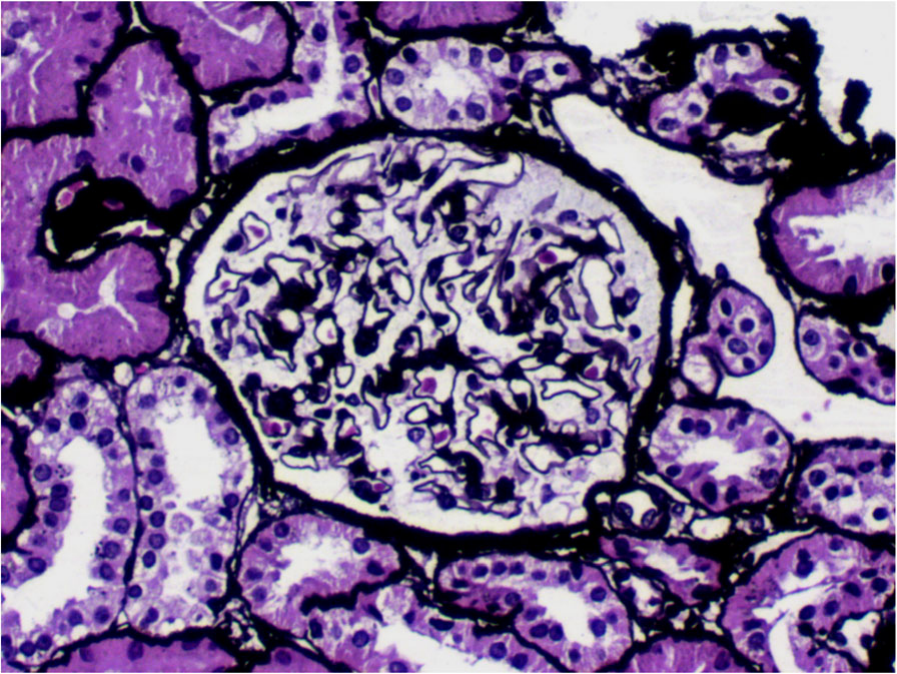
Kidney biopsy in light microscopy of the patient (PASM staining, magnification, ×200). Abbreviations: PASM = periodic Schiff-methenamine

Light microscopy showed previously unapparent proliferation of glomerular mesangial cells and mild segmental increases in the mesangial matrix (Fig.1). There was no hypertrophy of the juxtaglomerular apparatus or significant interstitial fibrosis or tubular trophy. Electron microscopy revealed diffuse effacement of the foot processes and no other significant ultrastructural abnormalities. Immunofluorescence demonstrated no deposition of immunoglobulins (IgG, IgA, and IgM) or complement (C3, C4, and C1q), and the kappa and lambda chains were also negative. The renal pathology was consistent with the development of minimal lesions. The patient was treated with prednisone (60 mg/d) and achieved complete remission after 3 weeks of treatment. Prednisone was tapered and administered for a total of 19 months before drug withdrawal. However, the level of serum potassium was lower than normal, despite taking potassium agents (KCl: 1.5 g/d).

One year ago, the patient experienced a relapse coinciding with an upper respiratory infection and was admitted to our hospital. He had no particular past history. His parents were not consanguineous. His father had passed away without a confirmed cause of death. The patient’s elder brother was prone to hypokalemia after sweating. The mother and sister were both healthy. Physical examination revealed a sitting blood pressure of 109/72 mmHg. Cr and alb were 56 μmol/L and 45.3 g/L, respectively. The amount of 24 h protein excretion was 3.98 g. Blood electrolytes, angiotensin-II, 24 h urine electrolytes, and the random UK/UCr ratio are presented in Table 1. Rennin, aldosterone, and angiotensin-I levels were normal. The serum potassium was 3.46 mmol/L after taking 4 g/d KCl and 80 mg/d spironolactone, which reached a normal range after the intake of magnesium aspartate.

**Fig. 2:**
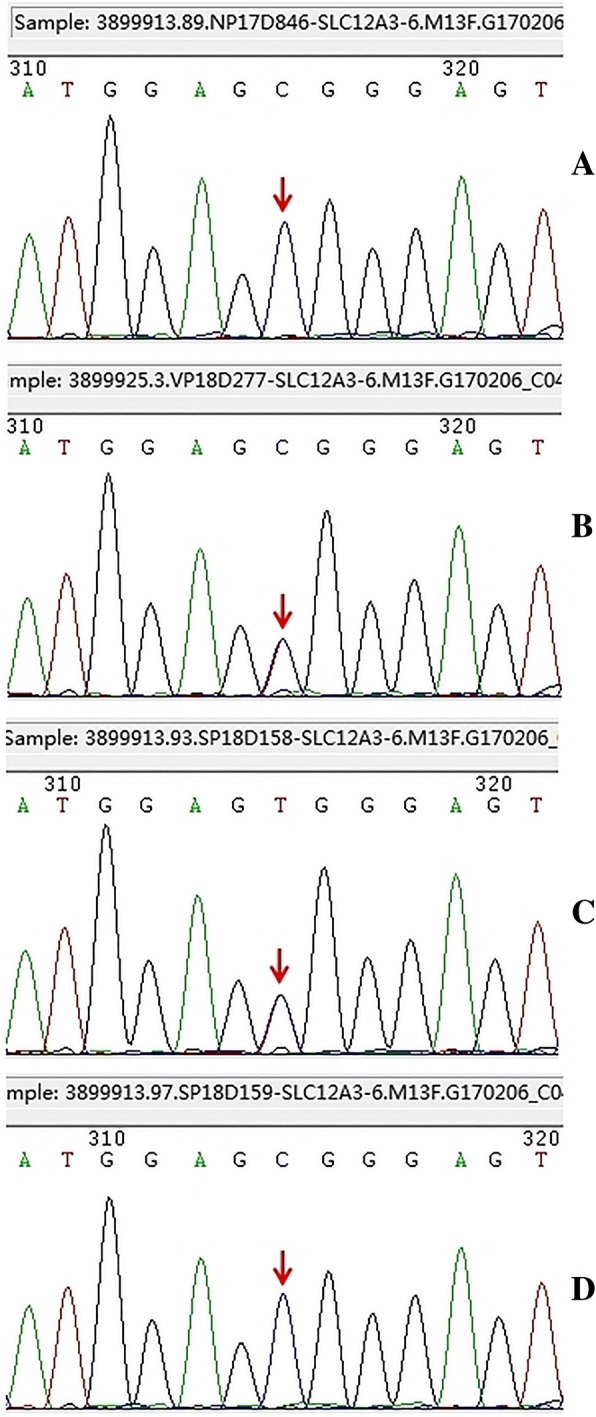
Genetic analysis of the SLC12A3 gene. (**a**: Patient, **b**: Mother, **c**: Sister, **d**: Brother)

Genetic analysis was performed after informed consent had been obtained from the patient and his family members. KingMed Diagnostics was responsible for the sequence analysis. The method of sequencing was as below. Total DNA was extracted from blood peripheral leukocytes by the QIAamp Blood DNA Mini Kit (QIAGEN, America). Premier 5 software was used to design primers specific to coding regions in SLC12A3, CLCNKB, CLCNKA, SLC12A1, KCN1 and BSND, which were amplified by polymerase chain reaction and directly sequenced. DNA sequencing results were compared with the normal sequence (NM_000339.2, NM_000085.4, NM_004070.3, NM_000338.2, NM_000220.2, NM_057176.2) to identify any potential mutations. Databases of ESP6500, 1000 Genomics, HGMD, ClinVar, UniProt and dbSNP were searched to predict the location of disease-causing genes. SIFT, PolyPhen-2, LRT, Mutation Taster, Mutation Assessor, FATHMM, GERP, PhyloP and SiPhy softwares were employed to predict the pathogenicity of putative missense mutations. Net Gene2 Server and AUGUSTUS softwares were used to predict the pathogenicity of Splicing change. Sequence analysis of the SLC12A3 gene revealed a homozygous missense mutation in exon 6 (c.841 T > C), which is likely to cause an arginine substitution (for tryptophan) at codon 281 (p.Trp281Arg) (Fig.2), and two heterozygous mutations in exon 15 (c.1568C > A; c.1551C > G) of the CLCNKA gene (Table 2). The mutations in SLC12A3 and CLCNKA have not been reported in literature thus far. Bioinformatics software predicted the homozygous missense mutation of SLC12A3 had larger possibility of disease, and a new heterozygous missense mutation of CLCNKA (c.1568C > A in exon 15, p.Thr523Asn) was probably damaging and a heterozygous synonymous mutation of CLCNKA (c.1551C > G in exon15, p.Pro517=) had no obvious effect on mRNA splicing. No mutations were detected in any of the exons and exon–intron boundaries in the CLCNKB, SLC12A1, KCN1 and BSND genes.

**Table 2:** Mutations related to GS and BS

Family member	Gene mutation	Exon	Nucleotide change	Amino acid change	Predicted Effect	HGVS ID
Patient	SLC12A3	6	c. 841 T > C	p. (Trp281Arg)	Homo, Missense	NM_000339.2
	CLCNKA	15	c. 1568C > A	p. (Thr523Asn)	Het, Missense	NM_004070.3
	CLCNKA	15	c. 1551 C > G	p. (Pro517=)	Het, Synonymous	NM_004070.3
Mother	SLC12A3	6	c. 841 T > C	p. (Trp281Arg)	Het, Missense	NM_000339.2
	CLCNKA	15	c. 1551 C > G	p. (Pro517=)	Het, Synonymous	NM_004070.3
Sister	SLC12A3	6	c. 841 T > C	p. (Trp281Arg)	Het, Missense	NM_000339.2
Brother	SLC12A3	6	c. 841 T > C	p. (Trp281Arg)	Homo, Missense	NM_000339.2
	CLCNKA	15	c. 1568C > A	p. (Thr523Asn)	Het, Missense	NM_004070.3

*Homo* Homozygous mutation, *Het* Heterozygous mutation

## Discussion and conclusions

Gitelman syndrome (GS) and Bartter syndrome (BS) are autosomal recessive disorders with biochemical features including hypokalemic, hypochloremic metabolic alkalosis associated with high plasma renin activity, and high aldosterone concentrations [4]. The key difference between the two syndromes is the level of urinary calcium excretion, which is low in GS but high in Bartter syndrome. However, some patients with type 3 BS have normal urinary calcium excretion levels [4]. Lin et al. reported that patients with molecularly-proven GS have severe hypokalemia and normal serum magnesium and urinary calcium excretion levels [5]. Hence, a genetic diagnosis is very important in the distinction between GS and BS.

The molecular defects in chloride reabsorption in BS and GS originate at different sites of the nephron. The transport defects for BS occur at the thick ascending limb (TAL) of the loop of Henle, whereas the defect in GS resides at the distal convoluted tubule (DCT). The present patient showed biochemical abnormalities including hypokalemia, hypochloremic, low excretion of urinary calcium, and increased plasma angiotensin-2 activity. Genetic analysis indicated the patient was the carrier of a new homozygous missense mutation in exon 6 (c.841 T > C) of the SLC12A3 gene with a greater possibility of disease. Therefore, it is indisputable that the patient was diagnosed with GS. The patient has two mutations in CLCNKA, including a missense mutation (c.1568C > A) that is relevant to type IVb Bartter syndrome. However, type IVb BS was caused by double mutations in the CLCNKA and CLCNKB genes. Moreover, no diseases caused by CLCNKA gene mutations in humans have been reported [6], although a mild diabetes insipidus has been detected in CLCNKA knockout mice [7]. Thus, there had no evidence to support a diagnosis of BS. However, whether the CLCNKA gene mutations in humans cause disease needs further research.

To our knowledge, Gitelman syndrome patients are largely asymptomatic. However, there is extreme inter- and intra-familiar phenotype variability and significant heterogeneity in onset age in GS [5, 8]. It has been suggested that males manifest a more severe phenotype and have a much earlier age of onset than females, which has been attributed to the absence of female sex hormones that may attenuate the loss of NCCT function [8, 9]. Furthermore, patients with GS do not classically present with proteinuria. The few renal biopsies available most often show hyperplasia of the juxtaglomerular apparatus, with no glomerular and no tubular abnormalities. However, 6 patients with genetically proven GS did present with proteinuria with preserved renal function upon retrospective analysis [10]. Tseng found seven male patients developed chronic kidney disease (CKD), five patients with stage III and two patients with stage IV, six of which underwent renal biopsy. Biopsies revealed chronic tubulointerstitial nephritis in favor of hypokalemic nephropathy [11]. There are some case reports describing patients with Gitelman syndrome, which has progressed to end-stage renal disease [12, 13]. These reports suggest volume depletion and recurrent episodes of prerenal acute renal failure are contributing factors in the development of progressive renal insufficiency. Isolated cases of focal segmental glomerulosclerosis (FSGS) and C1q nephropathy in Gitelman patients have been reported as well [14, 15]. Devuyst also found that GS was related to glomerular proteinuria and abnormalities of the glomerular basement membrane (GBM) [16]; defects of the GBM and podocytes were also detected in the Slc12a3 knockout mouse model [17]. These studies suggest that the association between GS and secondary changes of the kidney is probably not coincidental.

Possible mechanisms could be considered explaining the changes GS patient kidneys. One reason is that chronic potassium depletion could increase the proximal tubular cell production of ammonia and impair urinary concentrating ability by decreasing the distal nephron responsiveness to vasopressin [18, 19]. In keeping with this, Reungjui detected mild proteinuria in hypokalaemic rats given a normal or moderately low potassium diet, with or without hydrochlorothiazide [20]. Another point to consider is that the chronic activation of the renin angiotensin-aldosterone pathway, leading to increased systemic and local levels of angiotensin-II and renin, may in turn cause podocyte lesions. Angiotensin-II could lead to proteinuria through haemodynamic and non-haemodynamic mechanisms involving vascular endothelial growth factor (VEGF) and transforming growth factor (TGF)-β1 [21]. One case of GS was combined with a severe, non-apoptotic detachment of podocytes, in which the patient showed declining nephrin expression within the kidney [22]. The renal pathology of the patient was consistent with minimal lesions. Minimal disease and FSGS are podocyte diseases. Whether there may be a link between GS and podocyte injury requires further research.

In conclusion, we report a patient with Gitelman syndrome with hypokalemia and proteinuria. This case is the first to report a homozygous mutation in the 841th nucleotide of exon 6 on the SLC12A3 gene (p.Trp281Arg), which may cause Gitelman syndrome. At the same time, this report might stimulate interest in discussing the relationship between different mutations in the SLC12A3 gene and renal pathology.
